# Trastuzumab/paclitaxel-induced pneumonitis in breast cancer

**DOI:** 10.5339/qmj.2024.qitc.12

**Published:** 2024-03-25

**Authors:** Mustafa A. Al-Tikrity, Theeb Osama sulaiman, Mohammed Gaber, Aasir M. Suliman, Aisha Hussain

**Affiliations:** 1 Hamad General Hospital, Hamad Medical Corporation, Doha, Qatar Email: maltikrity@hamad.qa; 2 Center for Cancer Care and Research, Hamad Medical Corporation-NCCCR, Doha, Qatar

**Keywords:** Interstitial Lung Disease (ILD), Drug-induced pneumonitis, Paclitaxel, Trastuzumab, Breast cancer treatment

## Background

Interstitial lung disease (ILD) is a group of lung diseases that affect the interstitium. An inflammatory response occurs due to alveolar epithelial cell injury.^1^ Paclitaxel and trastuzumab, commonly used in breast cancer, can rarely lead to ILD, including pneumonitis.

## Case Presentation

A 51-year-old woman with a history of stage IA invasive ductal carcinoma of the right breast underwent a mastectomy. She started adjuvant treatment with paclitaxel/trastuzumab chemotherapy and radiotherapy. Eight weeks later, she had acute shortness of breath, cough with mild yellowish sputum, and pleuritic chest pain.

**Figure 1.**
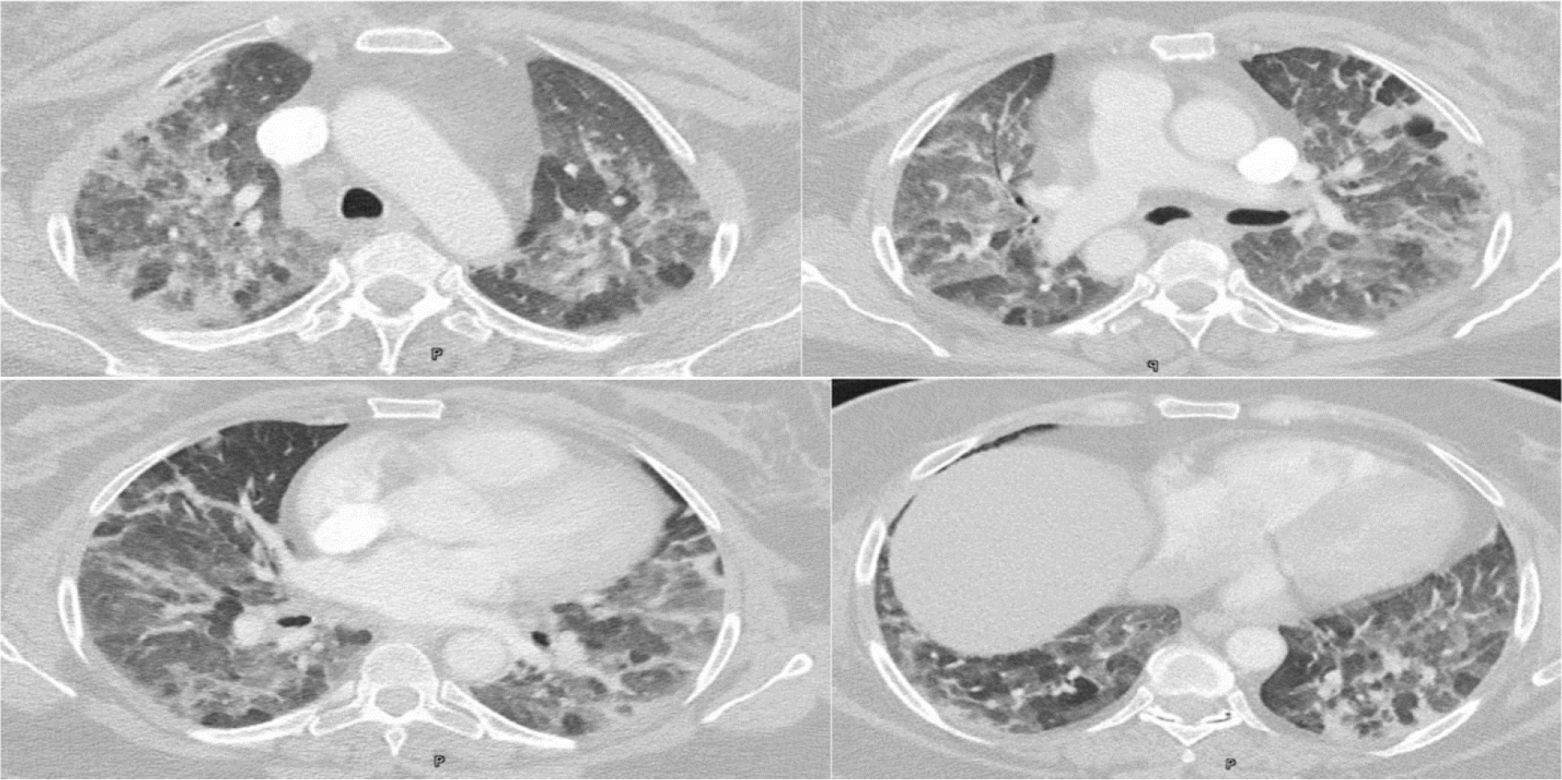
Images showing diffuse ground-glass opacities involving all lung lobes, with areas of small consolidation in the subpleural area of the right and left upper lobes.

**Figure 2.**
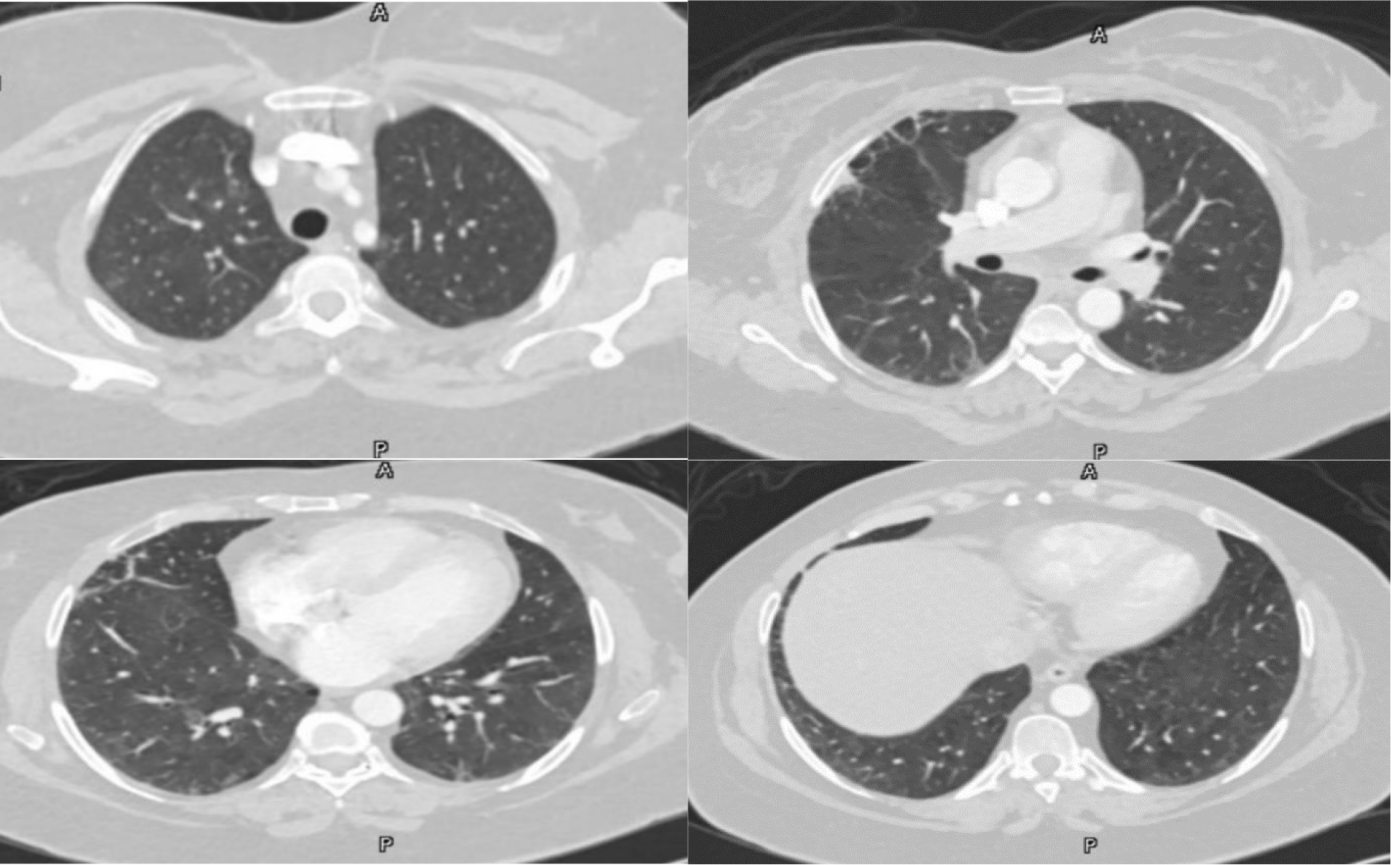
Significant regression of the previously noted diffuse bilateral lung infiltrations with minimal residual faint ground-glass opacities.

She was presented with tachypnea, tachycardia, and hypoxia. Pulmonary embolism was ruled out by a CT pulmonary angiogram, which showed diffuse ground-glass opacities in the lungs. The initial viral nasopharyngeal swab including Covid-19 was negative (Figure 1). The patient was admitted, and initial blood investigations showed low inflammatory markers. Bronchoscopy did not show significant findings, and bronchoalveolar lavage (BAL) and wash were negative for various pathogens, except for respiratory syncytial virus, which did not reveal such a picture. The diagnosis of drug-induced pneumonitis due to trastuzumab and/or paclitaxel was considered, and treatment for acute pneumonitis was initiated with prednisolone, resulting in significant clinical improvement and reduced oxygen requirements within 3 days. After discharge, the patient continued follow-up in the clinic. Trastuzumab was permanently discontinued, and follow-up chest CT 4 weeks later showed significant regression of the infiltrates. She remained asymptomatic and clinically stable (Figure 2).

Previous literature reported some incidents of trastuzumab- or paclitaxel-induced pneumonitis.^2^ This patient’s pulmonary symptoms were related to the toxicity of such medications in breast cancer treatment.

## Conclusion

It is essential to be aware of the rare occurrence of pneumonitis as a side effect of paclitaxel/trastuzumab. Given the overlap of clinical signs with other lung conditions, proper investigation, including microbial testing from bronchoscopic lavage/wash, is important in addition to early treatment.

## Conflict of Interest

The authors declare no conflict of interest regarding this case report.
